# Salivary Flow Rate in Patients with Kidney Failure on Hemodialysis—A Systematic Review and Meta-Analysis

**DOI:** 10.3390/jcm14176108

**Published:** 2025-08-29

**Authors:** Parinaz Mohammadi, Casper P. Bots, Henk S. Brand

**Affiliations:** 1 Department of Oral Biochemistry, Academic Centre for Dentistry Amsterdam (ACTA), 1081 LA Amsterdam, The Netherlands; 2 Dutch Institute for Salivary Research & the Oral Care Clinic, 3752 NA Bunschoten, The Netherlands; casperbots@gmail.com; 3 Department of Biochemistry, Dental Faculty, Carol Davila University of Medicine and Pharmacy, 041292 Bucharest, Romania

**Keywords:** Saliva, hemodialysis, kidney failure

## Abstract

**Background/Objectives:** During kidney failure, chronic hemodialysis therapy (HD) is required to replace lost renal function, and patients on regular HD frequently report xerostomia. This systematic review and meta-analysis aims to compare salivary flow rates between patients with kidney failure on HD and healthy controls and to evaluate acute changes in salivary secretion before and after a dialysis session. **Methods:** A systematic review was conducted in accordance with PRISMA guidelines. PubMed, Web of Science, and Embase were searched for observational studies quantifying salivary flow rates in adult patients with kidney failure on chronic hemodialysis versus healthy controls or pre- versus post-dialysis. Data on salivary flow rates were extracted and stratified by subtype (whole or gland-specific) and condition (stimulated or unstimulated), along with key study characteristics including participant demographics, saliva collection methods, and dialysis duration. Study quality was appraised using NHLBI tools and categorized as poor, fair, or good. Where ≥2 homogeneous datasets existed, random-effects meta-analyses (α = 0.05) were performed to estimate mean differences (95% CI) for each salivary parameter; heterogeneity was evaluated via I^2^. **Results:** A total of 20 studies (13 cross-sectional, 7 before-after) met inclusion, of which 17 studies (with a total of 1224 HD patients and 548 controls) were meta-analyzed. Compared with controls, HD patients showed lower secretion rates of unstimulated whole saliva (UWS: MD −0.11 mL/min; 95% CI −0.20 to −0.02; I^2^ = 94%) and stimulated whole saliva (SWS: MD −0.77 mL/min; 95% CI −0.94 to −0.60; I^2^ = 92%), whereas stimulated parotid saliva (SPS) did not differ significantly (MD −0.08 mL/min; 95% CI −0.77 to 0.60; I^2^ = 96%). In before-after analyses, both UWS (MD +0.15 mL/min; 95% CI 0.02–0.28; I^2^ = 90%) and SWS (MD +0.20 mL/min; 95% CI 0.14–0.26; I^2^ = 0%) increased immediately post-HD. **Conclusions:** Despite methodological challenges and population heterogeneity, the evidence indicates salivary hypofunction in HD patients and improvement after hemodialysis. The magnitude of these effects seems influenced by underlying comorbidities (notably diabetes), HD duration, and methodological factors. Since saliva is of major importance to maintaining good oral health, recognizing and managing dry mouth should therefore be part of the comprehensive care of patients with kidney failure.

## 1. Introduction

Chronic kidney disease (CKD) is a significant global health concern, affecting approximately 10–13% of the population worldwide [1,2]. It is a progressive condition characterized by impaired renal function leading to the accumulation of metabolic waste products, fluid imbalances, and disturbances in homeostasis. The Global Burden of Disease studies identify CKD as the third fastest-growing cause of mortality worldwide, highlighting its increasing impact on public health [3]. CKD is defined by a reduction in glomerular filtration rate (GFR) to <60 mL/min/1.73 m^2^ persisting for a period of at least three months. Several etiologies contribute to CKD, with diabetes mellitus, hypertension, and glomerulonephritis being the most prevalent. The disease may progress through five stages, culminating in kidney failure, where GFR falls below 15 mL/min/1.73 m^2^ or dialysis is required [1,4]. At this stage, kidney function typically decreases to 5–10% of normal capacity.

Patients with kidney failure require renal replacement therapy, which typically involves either dialysis or transplantation. Kidney transplantation is considered the most durable form of treatment to restore normal kidney function and homeostasis. However, due to the limited number of donors available and complications that may occur with immunosuppressive therapy and potential biological rejection, most patients with kidney failure rely on chronic dialysis treatment. Dialysis treatment includes two main types: peritoneal dialysis and hemodialysis, with hemodialysis being the most commonly used method. In hemodialysis the blood is filtered by an artificial kidney, whereas in peritoneal dialysis the peritoneal membrane is used. Hemodialysis may also be temporarily employed in acute conditions such as acute kidney injury or severe electrolyte disturbances, with a therapy duration usually limited to three months [5]. Therapy lasting more than three months is considered chronic hemodialysis, with a standard HD schedule of 4 h 3 times per week. Because of the reduced ability of the kidneys to excrete fluid, patients are recommended to restrict their daily fluid intake to 500 mL to prevent serious systemic complications [6].

Besides systemic effects, hemodialysis has also been associated with oral health complications. Xerostomia, a subjective sensation of dry mouth, is highly prevalent among hemodialysis patients, affecting approximately 33–76% of this population [7]. Although fluid restriction and medications contribute to xerostomia, recent research indicates that hemodialysis itself may have a direct impact on salivary flow rate and saliva composition [8].

Saliva is essential for maintaining oral health through several protective functions, including lubrication, acid buffering, maintenance of tooth integrity, and antimicrobial activity [9]. Reduced salivary secretion, known as hyposalivation, significantly increases the risk of oral diseases such as caries, erosion, oral candidiasis, mucositis, and ulcerations, severely impairing patients’ quality of life [10].

Previous studies have reported an association between hemodialysis and a reduced salivary flow rate. This study aims to provide a systematic review of the literature regarding the effects of hemodialysis treatment on the salivary flow rate in patients with kidney failure. Specifically, it compares salivary secretion of patients with kidney failure on hemodialysis and healthy controls using cross-sectional studies and evaluates acute salivary changes due to hemodialysis sessions through before-after study designs.

## 2. Methods

### 2.1. Search Strategy

A systematic review of the literature was approved by the Internal Ethical Review Board of the Academic Centre for Dentistry Amsterdam under protocol number 2025-38934 and conducted following the PRISMA (Preferred Reporting Items for Systematic Reviews and Meta-analysis) guidelines [11]. Relevant studies were retrieved from the electronic databases PubMed, Web of Science, and Embase using a comprehensive search strategy applied on 17 July 2025 (see Appendix A). Studies were screened by title and abstract based on the predefined eligibility criteria. Full-text articles of potentially eligible studies were subsequently retrieved and reviewed for final inclusion. The selection process was independently conducted by two reviewers, with disagreements resolved through discussion.

Data extraction was performed on eligible studies for relevant outcomes. Results were stratified according to the type of saliva collected (whole saliva or saliva from specific salivary gland(s)) and whether salivary secretion had been stimulated during collection or not. A meta-analysis of homogenous data were conducted if sufficient data were available.

### 2.2. Inclusion and Exclusion Criteria

Studies eligible for inclusion were observational studies with a control group: specifically case series, case-control, cohort, cross-sectional, or before-after studies. The outcome of interest was salivary flow rate (SFR), reported in quantitative measurements. Only studies in the English language were included. The population consisted of adult participants with kidney failure undergoing chronic hemodialysis; if kidney failure was not explicitly stated, patients on hemodialysis for more than six months were assumed to have chronic kidney disease. No changes in participants’ general health status or medication regimens during the study period were required. Studies that focused exclusively on pediatric populations were excluded.

The research questions were structured using the PECO framework—Population; Exposure; Comparator; and Outcome [12].

For cross-sectional studies comparing patients on chronic hemodialysis with healthy controls, the following PECO criteria were used:

P: Patients with kidney failure undergoing chronic hemodialysis

E: Chronic hemodialysis therapy

C: Healthy subjects

O: Salivary flow rate in mL/min

For studies comparing the salivary flow rate before and after a dialysis session, the following PECO criteria were used:

P: Patients with kidney failure undergoing chronic hemodialysis

E: After hemodialysis

C: Before hemodialysis

O: Salivary flow rate in mL/min

### 2.3. Data Collection

The following data were extracted from the included studies: study design, salivary flow rate, number of participants, mean age and sex of participants, duration of hemodialysis treatment, excluded confounding factors, restriction of oral activity before measurement, method to stimulate saliva secretion rate, and collection method. Results expressed in mg/min were converted to mL/min assuming 1 g = 1 mL. When saliva was collected at several moments in cross-sectional studies, pre-dialysis data were used for the meta-analysis.

### 2.4. Quality Assessment

The quality of included studies was assessed using the National Heart, Lung, and Blood Institute (NHLBI) quality assessment tools [13]. For studies with a cross-sectional design or studies including a cross-sectional group, the Quality Assessment Tool for Observational Cohort and Cross-Sectional Studies was used. For the studies with a before-after design, the Quality Assessment Tool for Before-After (Pre-Post) Studies With No Control Group was used.

Each included study was evaluated on the assigned criteria of the assessment tool, resulting in an overall quality rating. Studies received one point when a question was answered with ‘yes’ or ‘NA’, and no points were assigned when a question was answered with ‘no’ or ‘NR’. This resulted in a total score with a maximum of 14 points for cross-sectional studies and a maximum of 12 points for the before-after studies without a control group. The overall quality rating was given on the basis of the total number of obtained points. For the cross-sectional studies, the following rating was used: 0–5 points = poor, 6–9 points = fair, and 10–14 points = good. For the before-after studies, the grading was as follows: 0–4 points = poor, 5–8 points = fair, and 9–12 points = good. Because the NHLBI quality assessment tool does not prescribe numeric thresholds for overall study ratings, the abovementioned scoring system was adopted, consistent with approaches used in prior reviews [14,15,16].

### 2.5. Meta-Analysis

A meta-analysis was conducted to quantify the difference in salivary flow rates of patients with kidney failure before and after hemodialysis, as well as between patients with kidney failure and healthy controls. Specifically, salivary flow rates for stimulated whole saliva (SWS), unstimulated whole saliva (UWS), and stimulated parotid saliva (SPS) were compared between hemodialysis patients and healthy controls. Additionally, changes in unstimulated and stimulated whole salivary flow rates before and after a hemodialysis session were assessed in a separate meta-analysis. This resulted in five distinct pooled datasets categorized by study design and saliva collection method: UWS cross-sectional, SWS cross-sectional, SPS cross-sectional, UWS before-after, and SWS before-after.

A random-effects model was applied due to the anticipated high level of heterogeneity across studies. I^2^ values greater than 50% indicated substantial heterogeneity. The overall effect was assessed using Z-statistic, with a *p*-value below 0.05 considered statistically significant. Results were reported as mean differences with 95% confidence intervals (CIs).

## 3. Results

### 3.1. Study Selection

**Figure 1:**
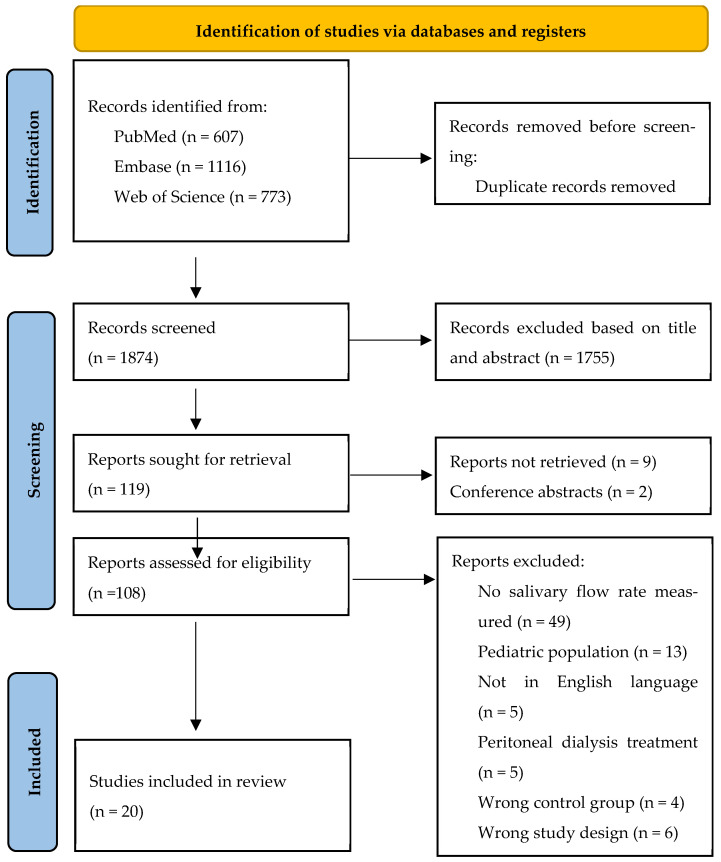
PRISMA flow diagram illustrating the study identification and selection process for inclusion.

The search strategy resulted in a total of 2113 records retrieved from three databases: PubMed (n = 607), Embase (n = 1116), and Web of Science (n = 773). After removing duplicates, 1874 unique records remained for screening. From the initial screening, 119 articles were selected for full-text review. During this stage, 9 publications had to be excluded because they could not be retrieved. Following eligibility assessment of the full texts, 20 studies were ultimately included in this systematic review. Reasons for exclusion during the full-text review phase are provided in the PRISMA flow diagram (Figure 1). The most common reason for exclusion was that salivary flow rate was not measured as an outcome. Other reasons for exclusion were related to an inappropriate study design, irrelevant population, publications in languages other than English, or other treatments than hemodialysis.

### 3.2. Quality Assessment

The methodological quality of the included studies was evaluated using the NHLBI quality assessment tools for Observational Cohort and Cross-Sectional Studies and Before-After (Pre-Post) Studies With No Control Group [13]. Studies were assigned an overall quality rating of “good,” “fair,” or “poor” based on predefined scoring thresholds.

An overall quality rating of “good” was achieved in two studies [17,18]. These studies reported and controlled for confounding factors, provided adequate justification for sample sizes, and included comprehensive methodological details.

In contrast, two studies received a “poor” quality rating [19,20]. Major limitations in these studies included insufficient reporting on study population characteristics, selection procedures, and saliva collection conditions. These studies were excluded from meta-analysis.

The remaining 16 studies were rated as “fair,” reflecting moderate methodological limitations but overall sufficient quality for inclusion. Common limitations among these studies included inadequate sample size justification (12 of 16 studies), insufficient details regarding participation rates (10 of 16 studies), and inadequate measurement or statistical adjustment for key confounding variables (11 of 16 studies).

One additional study [21] was excluded from the meta-analysis due to an incompatible study design. In this study, cross-sectional comparisons were made between separate patient groups assessed before (<3 days pre-dialysis) and after (<24 h post-dialysis) dialysis, rather than within-subject comparisons or cross-sectional comparisons with healthy controls.

Detailed results of the quality assessments, including scores for each specific criterion per study, are presented in Table 1 (cross-sectional studies) and Table 2 (before-after studies).

### 3.3. Study Characteristics

This review included a total of 20 studies published between 1977 and 2021, conducted in 13 different countries. The studies comprised 13 cross-sectional studies and 7 before-after studies. Among the cross-sectional studies, six studies compared hemodialysis patients with healthy controls, while one study made a comparison before and after a dialysis session in different patient groups [21]. Additionally, one cross-sectional study incorporated a before-after design in a small subgroup [28]. Among the before-after studies, two also included a cross-sectional comparison to healthy controls [27,32].

The characteristics of the studies included in each meta-analysis are presented in Table 3, Table 4, Table 5, Table 6 and Table 7, organized by saliva type (unstimulated whole, stimulated whole, or stimulated parotid) and study design (cross-sectional or before-after).

### 3.4. Study Population

The study populations primarily consisted of adults with kidney failure undergoing chronic hemodialysis therapy, with comparison to healthy controls in some studies. In three studies, kidney failure was not explicitly reported [29,30,35]; the study population was defined as patients undergoing regular hemodialysis therapy.

Several studies excluded patients with systemic diseases or therapies known to affect salivary flow [17,18,21,23,28,30,31,32,33,34,35,36]. There was a variation in whether patients with diabetes mellitus were included. Three studies explicitly excluded patients with diabetes mellitus [17,21,28], while four studies explicitly included diabetic patients [18,23,34,35]. Trzcionka et al. [32] stratified their population based on the presence of diabetes and/or hypertension. The remaining studies did not report excluding any systemic diseases or related medications in the study population.

Some studies also considered lifestyle factors, excluding smokers [30,31] and habitual drinkers [31]. The study of Yu et al. [36] only included patients experiencing mouth dryness in the four weeks prior to the study.

Several studies used a criterion for minimum dialysis duration, ranging from three months [33,35] to six months [23,29,30]. Trzcionka et al. [32] restricted the study population to patients over 40 years of age with kidney failure diagnosed at least 2 years prior to the study. Shetty et al. [29] stratified individuals based on dialysis duration.

### 3.5. Participant Age and Sex

Several studies used a criterion for minimum dialysis duration, ranging from three months [33,35] to six months [23,29,30]. Trzcionka et al. [32] restricted the study population to patients over 40 years of age with kidney failure diagnosed at least 2 years prior to the study. Shetty et al. [29] stratified individuals based on dialysis duration.

### 3.6. Treatment Details

The mean duration of dialysis of the study population was reported in 11 studies, ranging from one to six years [20,21,22,24,25,26,27,28,33,35,36]. Other studies did not report the mean duration of dialysis of the study group [17,18,19,23,30,31,32,34].

### 3.7. Saliva Collection Details

Stimulated whole saliva was collected in 11 studies, while unstimulated whole saliva was collected in 10 studies. Stimulated parotid saliva was collected in four studies [19,20,24,26], with one study also collecting unstimulated parotid saliva [20]. Additionally, stimulated and unstimulated submandibular saliva were collected in the latter study [20].

The majority of studies implemented a restriction of oral activity (such as eating) ranging from one to two hours prior to saliva collection [17,21,22,25,26,27,29,30,31,32,33,34,35,36]. In contrast, six studies did not report such a restriction period [18,19,20,23,24,28].

The cross-sectional studies employed variable moments of sampling; saliva was collected pre-dialysis [18,22,27,28,30,32], post-dialysis [21,25,26,27,28,29,32], or on the day between dialysis sessions [20]. Two studies did not report the moment of sampling in relation to the dialysis schedule [22,24].

#### 3.7.1. Unstimulated Whole Saliva

Unstimulated whole saliva was collected in 10 studies, with 4 studies comparing salivary flow rate before and after a dialysis session and 6 studies comparing the salivary flow rate of patients undergoing chronic hemodialysis therapy to that of healthy controls. The primary method of saliva collection was the spitting technique, which was employed in various studies [17,21,25,26,30,33,34,36]. Most studies instructed the participants to spit regularly. One study asked subjects to keep their saliva in their mouth for the entire period and then spit [36].

In addition to the spitting method, alternative collection techniques were used in a few studies. The draining technique was used by Gavaldá et al. [24], a method where participants lean forward and passively let saliva drip into a container. In the study of Tomás et al. [31], a cotton from a Salivette collection device was placed at the base of the tongue, and the adsorbed saliva was subsequently extracted by centrifugation.

Saliva collection periods ranged from 5 min [24,31,33,36] to 10 min [17,21,25,26,30,34] among studies that collected unstimulated whole saliva.

#### 3.7.2. Stimulated Whole Saliva

Stimulated whole saliva was collected in 11 studies, with 5 studies with a before-after study design and 9 with a cross-sectional design. Three studies had a mixed design [27,28,32], reporting both a cross-sectional group and a before-after comparison in the hemodialysis group.

The predominant method for saliva stimulation was mechanical stimulation, which was used in all studies except one [24], where gustatory stimulation was achieved by placing 1 mL of a 2% citric acid solution on the tongue. Mechanical stimulation was usually achieved with a piece of paraffin wax. Other mechanical stimulation methods included Saxton’s test, in which participants chewed on a folded gauze pad [28]; chewing on the cotton swab of a Salivette kit [18]; and chewing on a rubber sheet attached to dental floss [35].

In seven studies [22,23,25,27,29,32,33], participants were instructed to chew a piece of paraffin wax for five minutes. A five-minute collection duration was also used by Gavaldá et al. and Marques et al. [24,35], while shorter durations were reported by Postorino et al. [28] (2 min) and Limeres et al. [18] (45 s).

Chewing intensity was addressed in a few studies. In two studies, subjects were warned if they did not chew intensively enough [22,23]. Bots et al. [33] instructed participants to chew at their habitual pace. Postorino et al. [28] used the Saxon test, which intrinsically requires vigorous chewing. The remaining studies did not report specifications on chewing intensity.

Saliva was mainly collected using the spitting method. In some cases, alternative collection techniques were employed. In two studies [18,28], a weight-based absorption method was used, and another study [24] used the draining technique.

#### 3.7.3. Stimulated Parotid Saliva

Four studies collected stimulated parotid saliva [19,20,24,26], all using a (modified) Lashley cup placed directly over Stensen’s duct for saliva collection. Shannon et al. [19] used gustatory stimulation with grape-flavored hard candy. In two studies [24,26], a 2% citric acid solution was used for stimulation during a collection period of 5 min. In another study [20], a 3 mL stimulated saliva sample was obtained using a lemon lozenge. The collection period was timed to determine the salivary flow rate.

Kho et al. [26] specified that the stimulated parotid saliva collected during the first 2 min was discarded and that the citric acid solution was applied on the tongue at 30 s intervals. Gavaldá et al. [24] specified that 1 mL of a 2% citric acid solution was used for stimulation.

#### 3.7.4. Unstimulated Parotid Saliva

One study collected unstimulated parotid saliva [20], using a Lashley cup placed directly over Stensen’s duct. A 1 mL sample was collected without stimulation, with the collection period timed to determine the salivary flow rate.

#### 3.7.5. Submandibular Saliva

Epstein et al. [20] was the only study collecting submandibular saliva under both stimulated and unstimulated conditions. Submandibular saliva was collected with a modified Block-Broth-Man collector. A 1 mL sample was collected without stimulation, and a 3 mL stimulated sample was obtained using a lemon lozenge. The collection period was timed in order to determine the salivary flow rate.

### 3.8. Clinical Findings

#### 3.8.1. Unstimulated Whole Saliva

Detailed results for unstimulated whole salivary flow of studies included in the meta-analysis are presented in Table 3 (cross-sectional studies) and Table 4 (before-after studies).

All four studies comparing salivary flow before and after a dialysis session found a significantly higher salivary flow rate post-dialysis [21,33,34,36]. In one study [34], the mean post-dialysis salivary flow rate even exceeded the normal range and could be qualified as hypersalivation.

Four out of six studies reported a significantly lower unstimulated salivary flow in the hemodialysis group compared to healthy controls [17,25,26,30], and in one study the lower unstimulated salivary flow did not reach statistical significance [24]. One study reported a higher unstimulated salivary flow rate in the hemodialysis group, but this difference was not statistically significant [31].

#### 3.8.2. Stimulated Whole Saliva

Findings on stimulated whole salivary flow from the meta-analyzed studies are summarized in Table 5 (cross-sectional studies) and Table 6 (before-after studies).

All nine studies with a cross-sectional design comparing patients with kidney failure on hemodialysis with healthy controls reported a significantly lower stimulated salivary flow rate in the hemodialysis group [18,22,23,24,25,27,28,29,32].

Four studies examined changes in the stimulated salivary flow rate before and after a dialysis session, all reporting an increase in salivary flow rate post-dialysis. In three studies this increase was statistically significant [27,33,35], while in one study the increase did not reach statistical significance [28].

#### 3.8.3. Stimulated Parotid Saliva

Detailed results on stimulated parotid salivary flow of studies included in the meta-analysis are presented in Table 7 (cross-sectional studies).

In three studies, stimulated parotid salivary flow was significantly lower in hemodialysis patients than in healthy controls [20,24,26], whereas Shannon et al. [19] reported no significant difference between pre- and post-dialysis measurements in hemodialysis patients.

#### 3.8.4. Unstimulated Parotid Saliva

The unstimulated parotid salivary flow rate was lower in the hemodialysis group compared to healthy controls, but this finding was not statistically significant [20].

#### 3.8.5. Submandibular Saliva

Epstein et al. [20] investigated both stimulated and unstimulated submandibular saliva. The stimulated submandibular saliva rate was significantly lower in the hemodialysis group compared to healthy controls. Although unstimulated submandibular saliva was also lower in the hemodialysis group, this difference was not statistically significant.

### 3.9. Meta-Analysis

For the meta-analysis, five separate datasets were created according to study design and type of saliva collected. For the cross-sectional studies comparing the salivary flow rates of patients with kidney failure to healthy controls, three different types of saliva—unstimulated whole saliva (UWS); stimulated whole saliva (SWS); and stimulated parotid saliva (SPS)—were included in meta-analyses. For studies examining salivary flow rate in hemodialysis patients before and after a single dialysis session, meta-analyses were performed for unstimulated whole saliva as well as stimulated whole saliva. A random-effects model was used due to the high level of heterogeneity across studies.

### 3.10. Comparison Between Hemodialysis Patients and Healthy Controls

#### 3.10.1. Unstimulated Whole Saliva

**Figure 2:**
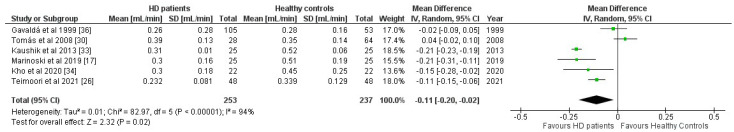
Forest plot of the meta-analysis of the comparison of the unstimulated whole salivary flow rate of patients with kidney failure on hemodialysis and healthy controls using a random-effects model.

In the UWS cross-sectional group, 6 studies were included for a total of 253 HD patients with a mean age of 55 years and 57% male participants (reported in 4 studies, n = 200). The control group consisted of 237 participants, with a mean age of 58 years and 54% male participants (reported in 4 studies, n = 190). One study did not report the mean age or sex distribution [25], while another study [26] stated that the control group was sex-matched but did not provide exact data. The salivary flow rate of hemodialysis patients was found to be significantly lower (*p* = 0.02), with a pooled mean difference of −0.11 mL/min (95% CI: −0.20, −0.02). However, substantial heterogeneity was observed among the included studies (χ^2^ = 82.97, df = 5, *p* < 0.00001; I^2^ = 94%), suggesting variability in study outcomes (Figure 2).

#### 3.10.2. Stimulated Whole Saliva

**Figure 3:**
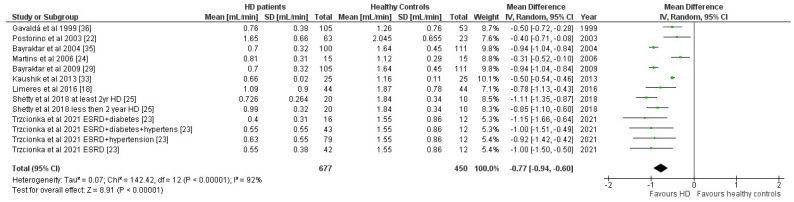
Forest plot of the meta-analysis of the comparison of the stimulated whole salivary flow rate of patients with kidney failure on hemodialysis and healthy controls using a random-effects model.

In the SWS cross-sectional group, 9 studies were included with a total of 677 HD patients in comparison to 450 healthy controls. Seven studies reported the mean age of participants, with a mean of 57 years in the HD group (n = 579) and 51 years in the control group (n = 344). Additionally, seven studies reported sex distribution in the HD group, resulting in a total of 65% male patients (n = 387), while six studies reported a total of 48% male participants in the control group (n = 300). Two studies did not provide data on mean age or sex [25,29], and another study only specified that the control group was sex-matched but did not provide specific data [18]. A pooled mean difference of −0.77 mL/min (95% CI: −0.94, −0.60; *p* < 0.00001) indicated a significantly lower salivary flow rate of stimulated whole saliva in HD patients in comparison to healthy controls. Substantial heterogeneity was present (χ^2^ = 142.42, df = 12, *p* < 0.00001; I^2^ = 92%), indicating considerable variation among the included studies (Figure 3).

#### 3.10.3. Stimulated Parotid Saliva

**Figure 4:**
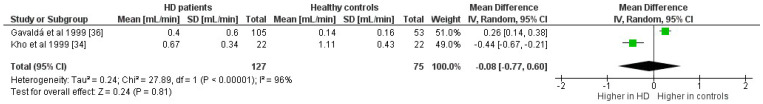
Forest plot of the meta-analysis of the comparison of the stimulated parotid salivary flow rate of patients with kidney failure on hemodialysis and healthy controls using a random-effects model.

In the SPS cross-sectional group, data from two studies were pooled to compare the stimulated parotid saliva secretion rate of 127 HD patients and 75 healthy individuals. The mean age of the HD group was 55 years, while the control group had a mean age of 48 years. Both studies reported the percentage of male participants in the HD group with a mean of 53%, and one study (n = 53) reported 55% males in the control group. The pooled mean difference of −0.08 mL/min (95% CI: −0.77, 0.60; *p* = 0.81) indicated no significant difference in stimulated parotid salivary flow between HD patients and healthy controls. A high heterogeneity was observed across studies (χ^2^ = 27.89, df = 1, *p* < 0.00001; I^2^ = 96%), suggesting substantial variability in study findings (Figure 4).

### 3.11. Before-After Comparison in Hemodialysis Patients

#### 3.11.1. Unstimulated Whole Saliva

**Figure 5:**
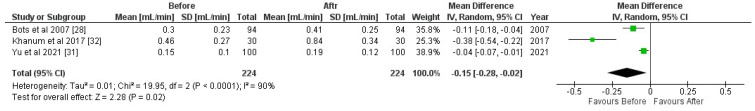
Forest plot of the meta-analysis of the comparison of the unstimulated whole salivary flow rate of patients with kidney failure before and after a hemodialysis session using a random-effects model.

The UWS before-and-after group included three studies (n = 224). One study [34] did not report mean age or sex distribution. The reported mean age of the other two studies (n = 194) was 60 years, and 67% were male participants. The pooled mean difference in UWS was −0.15 mL/min (95% CI: −0.28, −0.02; *p* = 0.02), indicating a statistically significant increase in salivary flow post-dialysis. However, a high level of heterogeneity was observed (χ^2^ = 19.95, df = 2, *p* < 0.00001; I^2^ = 90%), suggesting substantial variability across the included studies (Figure 5).

#### 3.11.2. Stimulated Whole Saliva

**Figure 6:**
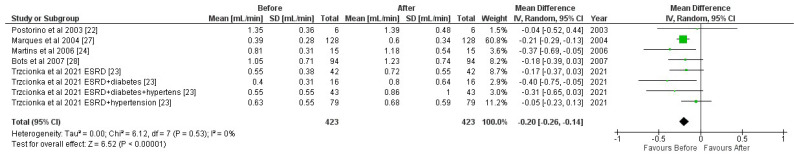
Forest plot of the meta-analysis of the comparison of the unstimulated whole salivary flow rate of patients with kidney failure before and after a hemodialysis session using a random-effects model.

The SWS before-and-after group comprised five studies (n = 423). One study [28] did not report participant age or sex. The other four studies (n = 417) reported a mean age of participants of 60 years, and 50% of the patients were male. The pooled mean difference in stimulated whole saliva (SWS) flow rate was −0.20 mL/min (95% CI: −0.26, −0.14; *p* < 0.00001), indicating a statistically significant increase in salivary flow post-dialysis. Heterogeneity among the included studies was low (χ^2^ = 6.12, df = 7, *p* = 0.53; I^2^ = 0%), suggesting minimal variability across study outcomes (Figure 6).

## 4. Discussion

This systematic review and meta-analysis of saliva secretion rates in patients on chronic hemodialysis (HD) produced two main findings. First, HD patients show significantly lower salivary flow rates than healthy controls for both unstimulated whole saliva (UWS) and stimulated whole saliva (SWS), whereas stimulated parotid saliva (SPS) is comparable between groups. Second, whole salivary flow (UWS and SWS) significantly increases within HD patients after a dialysis session. However, considerable heterogeneity exists across studies, possibly attributable to methodological variations and population differences.

A reduction in salivary flow rate in patients with kidney failure undergoing HD is constituted by a combination of intrinsic pathophysiological mechanisms and treatment-related influences. Mechanisms described in the literature include fluid restriction, uremia accumulation, glandular changes, systemic conditions, and polypharmacy.

In HD patients, strict oral fluid restriction is required to prevent volume overload [37]. Limited fluid intake combined with absent renal clearance leads to higher sodium and urea concentrations during the interdialytic interval, increasing plasma osmolality and inducing relative dehydration [38,39]. The resultant hyperosmolar plasma stimulates diffusion of uremic solutes into saliva, thereby stressing the salivary glands and suppressing secretion [19,40]. Fluid-restriction studies in healthy volunteers replicate this state of hyperosmolality with reduced salivary flow, confirming relative dehydration as a major cause of an HD-associated reduction in salivary flow rate [41,42]. Salivary flow rates clearly increase after dialysis, as clearance of urea and sodium eliminates the osmotic gradient and normalizes plasma osmolality [35,39,43].

There is increasing evidence that salivary function deteriorates as kidney failure progresses and the duration of HD treatment increases. Shetty et al. [29] observed progressively lower SWS in patients with longer HD history. In the study of Postorino et al. [28], histological examination of salivary glands from long-term HD patients showed fibrosis, acinar atrophy, and sclerosis. These changes are thought to result from chronic uremic toxicity and oxidative stress and are linked to reduced secretory capacity. In contrast, Bots et al. [33] found that salivary flow rates recovered after renal transplantation, indicating that the deficiency is reversible and reflects the uremic milieu rather than permanent dialysis-induced gland damage. Autonomic nervous system dysfunction has also been proposed as a contributor to a CKD-related reduction in salivary flow rate [44], but human studies have yet to confirm this neuropathic mechanism in patients with kidney failure.

The studies included in our review showed a wide age range of participants, from a mean age of ~35 years [26] to ~70 years [18]. Aging itself is correlated with reduced salivary flow, as structural and degenerative changes gradually reduce secretory tissue in all salivary glands [45]. A meta-analysis of 47 studies showed that unstimulated and chewing-stimulated whole saliva and submandibular/sublingual flow rates decrease with age, whereas parotid output remains largely preserved after adjusting for medication use [46]. However, the included studies involved an age-matched healthy control group, making it unlikely that the observed decreases in salivary secretion rate within the included studies are due to differences in age. However, the magnitude of the difference between hemodialysis patients and healthy volunteers could differ in absolute and percentage terms based on differences in age of the study populations studied.

Polypharmacy negatively affects salivary secretion rate. Most ESRD patients receive multiple drugs—especially antihypertensives; antidepressants; and antiemetics—with anticholinergic or sympatholytic activity that further suppresses salivary secretion.

Comorbidity or underlying etiology of kidney failure can significantly affect both baseline flow and response to HD. Trzcionka et al. [32] documented a significant increase in SWS post-HD in diabetic (non-hypertensive) patients, but no significant change in salivary flow in the subgroup of HD patients with hypertension. Similarly, Shetty et al. [29] reported the highest pre-HD flow and a non-significant post-HD change in non-diabetic hypertensive patients with kidney failure, although saliva flow remained subnormal compared to controls.

Diabetes mellitus seems to have a particularly large impact and is independently associated with reduced salivary flow [47]. Diabetes is an important factor because insulin deficiency reduces salivary flow [43]. A GFR < 50 mL/min has been shown to cause insulin resistance [48], exacerbating oral dryness. Diabetic HD patients report more severe xerostomia and thirst versus non-diabetics [49,50]. Studies that explicitly excluded HD patients with diabetes [17,28] still confirmed reduced SWS in HD patients, highlighting kidney failure itself as a contributing factor to salivary hypofunction.

This review has several limitations. There was substantial between-study heterogeneity, limiting the precision of pooled estimates. The SPS finding was based on data from only two studies and should therefore be interpreted with caution. Only English-language articles were included, and most studies concerned Western cohorts; therefore, the findings may not be fully generalizable to other geographic or cultural populations. On the other hand, a systematic review concluded that restricting systematic reviews to English-language publications appears to have little impact on the effect estimates and conclusions of systematic reviews for most medical topics [51].

UWS findings were highly technique dependent. The draining method used by Gavaldá et al. [24] showed no significant difference in comparison to controls, while studies that required frequent spitting often reported significant deficits. The spitting method refers to a technique originally described by Navazesh and Christensen to collect unstimulated whole saliva [52]. This method generally involves having the subject sit comfortably and lean their head slightly forward to pool saliva in their mouth, then periodically spitting the pooled saliva into a collection container. However, this technique is associated with (minimal) oral movement of the mouth, suggesting spitting may inadvertently stimulate saliva flow. Absorption techniques yielded mixed results: short cotton or gauze placements (45 s in Limeres et al. [18]; 2 min in Postorino et al. [28]) showed reductions, but the 5 min cotton protocol of Tomás et al. [31] did not. Introducing cotton into the mouth might also stimulate the salivary glands to some degree. In summary, this means that it is difficult to determine the actual unstimulated salivary secretion rate. However, this applies to both the hemodialysis patients and the healthy controls.

The SWS data were also confounded by heterogeneous procedures: most studies used chewing, yet the intensity varied from the vigorous Saxon test to relaxed “natural” chewing, and some protocols did not report details on chewing. Moreover, several studies did not adhere to the recommended collection period of 5 min for potentially suppressed salivary flow [53]. Sample processing may also have affected results; centrifuging samples before weighing [36] yielded lower salivary flow values than weighing unprocessed saliva [33].

Variations in patient selection criteria and baseline characteristics further limit the strength of the conclusions. The included studies varied in their inclusion/exclusion criteria regarding comorbid conditions (especially diabetes and hypertension) and use of xerostomia-inducing medications. Some studies tried to control for these factors, while others had more heterogeneous study populations, which may have influenced salivary flow outcomes. There were notable demographic imbalances in certain studies; for example, the HD group in one study was only ~31% male vs. 60–63% male in the control group [32], which is relevant given that females generally have lower UWS flow rates than males [54].

Selection bias in the study populations must also be considered. Not all investigations sampled the HD patient population in the same way, which may have influenced results. For instance, one study [36] required that participants report a history of dry mouth in the past four weeks as an inclusion criterion.

Xerostomia is often associated with a reduction in salivary flow rate, but the relationship is not perfectly linear. In patients with kidney failure, those with markedly low salivary output frequently report dry mouth, and indeed, studies have found that lower UWS flow rates tend to correspond with higher xerostomia scores and more thirst in the HD population [33]. Exceeding fluid restrictions to relieve thirst causes interdialytic weight gain. Interdialytic weight gain is associated with cardiovascular death and increased morbidity, such as ventricular hypertrophy and major adverse cardiac and cerebrovascular events [37].

A multimodal approach can reduce interdialytic weight gain (IDWG) and xerostomia: non-pharmacologic thirst modulators (such as sugar-free chewing gum, saliva substitutes, acupuncture, and transcutaneous electrical nerve stimulation) improve salivary flow and reduce fluid intake [55,56,57]. Low-sodium, home-delivered meals also reduce IDWG, thirst, and dryness scores [58], while mobile apps and telemonitoring enhance adherence to fluid restriction [55]. Individually tailored dialysis sessions, low-sodium dialysate, and biofeedback-controlled ultrafiltration further improve fluid balance and cardiovascular stability [27,49].

Reduced salivary flow has numerous negative consequences for oral function and oral health. Saliva plays a critical role in lubricating oral tissues, initiating digestion of food, buffering acids, and regulating microbial growth. Patients with chronic dry mouth often experience difficulty chewing, swallowing, and speaking, as well as altered taste sensation and chronic halitosis. In patients with kidney failure undergoing HD, a reduction in salivary flow rate may predispose individuals to oral infections such as candidiasis and may increase the risk of dental caries [59].

Dental clinicians should monitor the salivary flow rate of HD patients and counsel them on protective oral hygiene measures, the use of sialogogues, and frequent dental check-ups. Collaboration between dental professionals, nephrologists, and dietitians can improve treatment, for example, by emphasizing the importance of fluid restrictions and chewing sugar-free gum or lozenges to relieve oral dryness without increasing fluid intake [55,56].

Although our research shows that patients with kidney failure undergoing hemodialysis have a lower salivary secretion rate than healthy individuals, as indicated above in the Discussion, several factors may play a role in this. Further research into these factors is needed. In studies with sufficient numbers of subjects, hemodialysis patients could be stratified according to the cause of kidney failure to investigate the contribution of underlying pathology and medication. The addition of additional control groups in addition to healthy subjects could also be considered, such as patients taking similar antihypertensive medication for other reasons and patients with diabetes without ESRD. Such future research should also focus on standardization: adherence to established protocols for saliva collection, weight-based measurement of saliva secretion rates, controlled timing of collection of saliva relative to HD sessions and time of day, consistent protocols for stimulation of saliva secretion rates, detailed reporting of comorbidities and medication used, and implementation of blinded assessment.

## 5. Conclusions

Despite methodological challenges and population heterogeneity, the evidence of this review robustly indicates a reduction in salivary secretion rates in HD patients compared to healthy subjects and improvement of saliva secretion after a hemodialysis session. The magnitude of these effects and their persistence post-HD seem to be influenced by underlying comorbidities (notably diabetes), medication, duration of treatment with HD, and methodological factors.

Since saliva is of major importance to maintaining good oral health, recognizing and managing dry mouth should therefore be part of the comprehensive care of patients with kidney failure.

## Figures and Tables

**Table 1:** Quality assessment of cross-sectional studies.

Criteria	Bayraktar (2004) [22]	Bayraktar (2009) [23]	Epstein (1980) [20]	Gavaldá (1999) [24]	Kaushik (2013) [25]	Kho (1999) [26]	Kumar (2020) [21]	Limeres (2016) [18]	Marinoski (2019) [17]	Martins (2016) [27]	Postorino (2003) [28]	Shetty (2018) [29]	Teimoori (2021) [30]	Tomás (2008) [31]	Trzcionka (2021) [32]
1. Was the research question or objective in this paper clearly stated?	Yes	Yes	Yes	Yes	Yes	Yes	Yes	Yes	Yes	Yes	Yes	Yes	Yes	Yes	Yes
2. Was the study population clearly specified and defined?	No	Yes	No	Yes	No	Yes	Yes	Yes	Yes	Yes	Yes	No	Yes	Yes	Yes
3. Was the participation rate of eligible persons at least 50%?	NR	NR	NR	NR	NR	NR	NR	Yes	NR	NR	Yes	NR	NR	NR	NR
4. Were all the subjects selected or recruited from the same or similar populations (including the same time period)? Were inclusion and exclusion criteria for being in the study prespecified and applied uniformly to all participants?	NR	No	NR	NR	NR	Yes	Yes	Yes	Yes	Yes	NR	NR	No	Yes	Yes
5. Was a sample size justification, power description, or variance and effect estimate provided?	No	No	No	No	No	No	No	Yes	Yes	No	No	No	No	No	No
6. For the analyses in this paper, were the exposure(s) of interest measured prior to the outcome(s) being measured?	Yes	Yes	Yes	Yes	Yes	Yes	Yes	Yes	Yes	Yes	Yes	Yes	Yes	Yes	Yes
7. Was the timeframe sufficient so that one could reasonably expect to see an association between exposure and outcome if it existed?	No	No	No	No	No	No	No	No	No	Yes	No	No	No	No	Yes
8. For exposures that can vary in amount or level, did the study examine different levels of the exposure as related to the outcome (e.g., categories of exposure or exposure measured as a continuous variable)?	No	No	No	No	No	No	No	No	No	No	No	Yes	No	No	Yes
9. Were the exposure measures (independent variables) clearly defined, valid, reliable, and implemented consistently across all study participants?	Yes	Yes	Yes	Yes	Yes	Yes	No	Yes	Yes	Yes	Yes	Yes	Yes	Yes	Yes
10. Was the exposure(s) assessed more than once over time?	NA	NA	No	NA	NA	NA	NA	NA	NA	Yes	Yes	NA	NA	Yes	Yes
11. Were the outcome measures (dependent variables) clearly defined, valid, reliable, and implemented consistently across all study participants?	Yes	Yes	Yes	Yes	Yes	Yes	Yes	Yes	Yes	Yes	Yes	Yes	Yes	Yes	Yes
12. Were the outcome assessors blinded to the exposure status of participants?	NR	NR	NR	NR	NR	NR	NR	NR	NR	NR	NR	NR	NR	NR	No
13. Was loss to follow-up after baseline 20% or less?	NA	NA	NA	NA	NA	NA	NA	NA	NA	NR	Yes	NA	NA	NA	NR
14. Were key potential confounding variables measured and adjusted statistically for their impact on the relationship between exposure(s) and outcome(s)?	No	Yes	No	No	No	No	No	Yes	Yes	No	No	No	No	Yes	No
Total score	6	8	5	7	6	8	7	11	10	8	8	7	7	9	9
Quality rating	Fair	Fair	Poor	Fair	Fair	Fair	Fair	Good	Good	Fair	Fair	Fair	Fair	Fair	Fair

NHLBI Quality Assessment Tool for Observational Cohort and Cross-Sectional Studies.

**Table 2:** Quality assessment of before-after studies.

Criteria	Bots (2007) [33]	Khanum (2017) [34]	Marques (2015) [35]	Shannon (1977) [19]	Yu (2021) [36]
1. Was the study question or objective clearly stated?	Yes	Yes	Yes	No	Yes
2. Were eligibility/selection criteria for the study population prespecified and clearly described?	Yes	Yes	Yes	No	Yes
3. Were the participants in the study representative of those who would be eligible for the test/service/intervention in the general or clinical population of interest?	Yes	Yes	Yes	NR	No
4. Were all eligible participants that met the prespecified entry criteria enrolled?	NR	No	NR	NR	Yes
5. Was the sample size sufficiently large to provide confidence in the findings?	Yes	No	Yes	No	Yes
6. Was the test/service/intervention clearly described and delivered consistently across the study population?	Yes	Yes	Yes	Yes	Yes
7. Were the outcome measures prespecified, clearly defined, valid, reliable, and assessed consistently across all study participants?	Yes	Yes	Yes	Yes	Yes
8. Were the people assessing the outcomes blinded to the participants’ exposures/interventions?	No	NR	NR	NR	No
9. Was the loss to follow-up after baseline 20% or less? Were those lost to follow-up accounted for in the analysis?	NR	NR	NR	NR	NR
10. Did the statistical methods examine changes in outcome measures from before to after the intervention? Were statistical tests conducted that provided *p*-values for the pre-to-post changes?	Yes	Yes	Yes	NR	Yes
11. Were outcome measures of interest taken multiple times before the intervention and multiple times after the intervention (i.e., did they use an interrupted time-series design)?	No	No	No	No	No
12. If the intervention was conducted at a group level (e.g., a whole hospital, a community, etc.), did the statistical analysis take into account the use of individual-level data to determine effects at the group level?	NA	NA	NA	NA	NA
Total score	8	7	8	3	8
Quality rating	Fair	Fair	Fair	Poor	Fair

NHLBI Quality Assessment Tool for Before-After (Pre-Post) Studies With No Control Group.

**Table 3:** Characteristics of cross-sectional studies reporting unstimulated whole salivary flow rates.

Author, Year	Country	Groups (n), Mean Age and Sex	SFR HD	SFR C	Findings
Gavaldá et al. (1999) [24]	Spain	HD(105), 58.9 ± 14.9 y (50% m, 50% f) C (53), 55.7 ± 10.7 y (55% m, 45% f)	0.26 ± 0.28 mL/min	0.28 ± 0.16 mL/min	No significant difference in unstimulated whole salivary flow rates (*p* = 0.5)
Kaushik et al. (2013) [25]	India	HD(25), 44.4 ± 7.5 y (61% m, 39% f) C (25), not reported	0.31 ± 0.01 mL/min	0.52 ± 0.06 mL/min	Significantly lower unstimulated whole salivary flow rates in HD patients (*p* < 0.001)
Kho et al. (1999) [26]	Republic of Korea	HD (22), 34.7 ± 10.8 y (64% m, 36% f) C (22), 30.5 ± 7.9 y (sex not reported)	0.30 ± 0.18 mL/min	0.45 ± 0.25 mL/min	Significantly lower unstimulated whole salivary flow rates in HD patients (*p* < 0.05)
Marinoski et al. (2019) [17]	Serbia	HD(25), 54.9 ± 13.6 y (72% m, 28% f) C(25), 54.2 ± 12.7 y (64% m, 36% f)	0.30 ± 0.16 mL/min	0.51 ± 0.19 mL/min	Significantly lower unstimulated whole salivary flow rates in HD patients (*p* < 0.001)
Teimoori et al. (2021) [30]	Iran	HD (48), 53.5 ± 1.9 y (58% m, 42% f) C (48), 59.3 ± 1.6 y (58% m, 42% f)	0.232 ± 0.081 mL/min	0.339 ± 0.129 mL/min	Significantly lower unstimulated whole salivary flow rates in HD patients (*p* = 0.000)
Tomás et al. (2008) [31]	Portugal	HD (28), 64 ± 11 y (46% m, 54% f) C (64), 60 ± 11 (43% m, 57% f)	0.39 ± 0.13 mL/min	0.35 ± 0.14 mL/min	No significant difference in unstimulated whole salivary flow rates (*p* > 0.05)

SFR = salivary flow rate HD = patients with kidney failure on hemodialysis C = healthy controls.

**Table 4:** Characteristics of before-after studies reporting unstimulated whole salivary flow rates.

Author, Year	Country	Groups (n), Mean Age, and Sex	SFR BHD	SFR AHD	Findings
Bots et al. (2007) [33]	The Netherlands	Pre, Post HD (94), 56.4 ± 16.7 y (68% m, 32% f)	0.30 ± 0.23 mL/min	0.41 ± 0.25 mL/min	Significantly higher unstimulated whole salivary flow rates post-dialysis (*p* < 0.05)
Khanum et al. (2017) [34]	India	Pre, Post HD (30), not reported	0.46 ± 0.27 mL/min	0.84 ± 0.34 mL/min	Significantly higher unstimulated whole salivary flow rates post-dialysis (*p* = 0.000)
Yu et al. (2021) [36]	Taiwan	Pre, Post HD (100), 62.45 ± 11.09 y (66% m, 34% f)	0.15 ± 0.10 mL/min	0.19 ± 0.12 mL/min	Significantly higher unstimulated whole salivary flow rates post-dialysis (*p* = 0.006)

SFR = salivary flow rate BHD = before a hemodialysis session AHD = after a hemodialysis session.

**Table 5:** Characteristics of cross-sectional studies reporting stimulated whole salivary flow rates.

Author, Year	Country	Groups (n), Mean Age, and Sex	SFR HD	SFR C	Findings
Postorino et al. (2003) [28]	Italy	HD(63), 50.2 ± 13.8 y (60% m, 40% f) C (23), 46.0 ± 13.2 y (66% m, 34% f)	1.65 ± 0.66 mL/min	2.05 ± 0.66 mL/min	Significantly lower stimulated whole salivary flow rates in HD patients (*p* < 0.02)
Bayraktar et al. (2009) [23]	Turkey	HD (100), 46 ± 14 y (56% m, 44% f) C (111), 45 ± 18 y (41% m, 59% f)	0.70 ± 0.32 mL/min	1.64 ± 0.45 mL/min	Significantly lower stimulated whole salivary flow rates in HD patients (*p* < 0.001)
Gavaldá et al. (1999) [24]	Spain	HD (105), 58.9 ± 14.9 y (50% m, 50% f) C(53), 55.7 ± 10.7 y (55% m, 45% f)	0.76 ± 0.38 mL/min	1.26 ± 0.76 mL/min	Significantly lower stimulated whole salivary flow rates in HD patients (*p* < 0.001)
Kaushik et al. (2013) [25]	India	HD (25), 44.42 ± 7.53 y (61% m, 39% f) C (25), not reported	0.66 ± 0.02 mL/min	1.16 ± 0.11 mL/min	Significantly lower stimulated whole salivary flow rates in HD patients (*p* < 0.001)
Limeres et al. (2016) [18]	Portugal	HD (44), 69.8 ± 10.0 y (41% m, 59% f) C (44), 69.3 ± 10.0 y (sex not reported)	1.09 ± 0.90 mL/min	1.87 ± 0.78 mL/min	Significantly lower stimulated whole salivary flow rates in HD patients (*p* < 0.001)
Martins et al. (2006) [27]	Brazil	HD (15), 47.4 ± 9.73 y (67% m, 33% f) C (15), 39.8 ± 9.97 y (27% m, 73% f)	0.81 ± 0.31 mL/min	1.12 ± 0.29 mL/min	Significantly lower stimulated whole salivary flow rates in HD patients (*p* < 0.05)
Shetty et al. (2018) [29]	India	HD [dd 0.5–2 y] (20), not reported C (20), not reported	0.99 ± 0.32 mL/min	1.84 ± 0.34 mL/min	Significantly lower stimulated whole salivary flow rates in all subgroups of HD patients (*p* < 0.001) compared to controls. SFR HD [>2 y] < HD [0.5–2 y] (*p* < 0.05)
		HD [dd > 2 y] (20), not reported C (20), not reported	0.726 ± 0.264 mL/min	1.84 ± 0.34 mL/min	
Trzcionka et al. (2021) [32]	Poland	HD [R] (42), 67.21 y (60% m, 40% f) C (48), 52.71 y (31% m, 69% f)	0.55 ± 0.38 mL/min	1.55 ± 0.86 mL/min	Significantly lower stimulated whole salivary flow rates in all subgroups of HD patients (*p* < 0.001).
		HD [R + H] (79), 62.54 y (61% m, 40% f) C (48), 52.71 y (31% m, 69% f)	0.63 ± 0.55 mL/min	1.55 ± 0.86 mL/min	
		HD [R + D] (16), 70.16 y (63% m, 37% f) C (48), 52.71 y (31% m, 69% f)	0.40 ± 0.31 mL/min	1.55 ± 0.86 mL/min	
		HD [R + D + H] (43), 72.86 y (63% m, 37% f) C (48), 52.71 y (31% m, 69% f)	0.55 ± 0.55 mL/min	1.55 ± 0.86 mL/min	
Bayraktar et al. (2004) [22]	Turkey	HD (72), 45.05 ± 14.15 y (53% m, 47% f) C (50), 43.92 ± 18.80 y (48% m, 52% f)	0.69 ± 0.31 mL/min	1.64 ± 0.44 mL/min	Significantly lower stimulated whole salivary flow rates in HD patients (*p* < 0.001)

SFR = salivary flow rate HD = patients with kidney failure on hemodialysis C = healthy controls HD [dd 0.5–2 y] = HD patients with a total dialysis duration of 6 months–2 years HD [dd > 2 y] = HD patients with a total dialysis duration of >2 years.

**Table 6:** Characteristics of before-after studies reporting stimulated whole salivary flow rates.

Author, Year	Country	Groups (n), Mean Age, and Sex	SFR HD	SFR C	Findings
Bots et al. (2007) [33]	The Netherlands	Pre, Post HD (94), 56.4 ± 16.7 y (68% m, 32% f)	1.05 ± 0.71 mL/min	1.23 ± 0.74 mL/min	Significantly higher stimulated whole salivary flow rates post-dialysis (*p* < 0.05)
Marques et al. (2015) [35]	Brazil	Pre, Post HD (128), 56.2 ± 17.1 y (52% m, 48% f)	0.39 ± 0.28 mL/min	0.60 ± 0.34 mL/min	Significantly higher stimulated whole salivary flow rates post-dialysis (*p* < 0.001)
Martins et al. (2006) [27]	Brazil	Pre, Post HD (15), 47.4 ± 9.73 y (67% m, 33% f)	0.81 ± 0.31 mL/min	1.18 ± 0.54 mL/min	Significantly higher stimulated whole salivary flow rates post-dialysis (*p* < 0.05)
Postorino et al. (2003) [28]	Italy	Pre, Post HD (6), not reported	1.35 ± 0.36 mL/min	1.39 ± 0.48 mL/min	No significant difference in stimulated whole salivary flow rates before or after dialysis
Trzcionka et al. (2021) [32]	Poland	Pre, Post HD [R] (42), 67.21 y (60% m, 40% f)	0.55 ± 0.38 mL/min	0.72 ± 0.55 mL/min	Significantly higher stimulated whole salivary flow rates post-dialysis in all subgroups of HD patients (*p* < 0.001).
		Pre, Post HD [R + H] (79), 62.54 y (61% m, 40% f)	0.63 ± 0.55 mL/min	0.68 ± 0.59 mL/min	
		Pre, Post HD [R + D] (16), 70.16 y (63% m, 37% f)	0.40 ± 0.31 mL/min	0.80 ± 0.64 mL/min	
		Pre, Post HD [R + D + H] (43), 72.86 y (63% m, 37% f)	0.55 ± 0.55 mL/min	0.86 ± 1.00 mL/min	

SFR = salivary flow rate HD = patients with kidney failure on hemodialysis C = healthy controls HD [R] = HD patients diagnosed with kidney failure HD [R + H] = HD patients diagnosed with kidney failure and hypertension HD [R + D] = HD patients diagnosed with kidney failure and diabetes HD [R + D + H] = HD patients diagnosed with kidney failure, diabetes and hypertension.

**Table 7:** Characteristics of cross-sectional studies reporting stimulated parotid salivary flow rates.

Author, Year	Country	Groups (n), Mean Age, and Sex	SFR HD	SFR C	Findings
Gavaldá et al. (1999) [24]	Spain	HD (105), 58.9 ± 14.9 y (50% m, 50% f) C (53), 55.7 ± 10.7 y (55% m, 45% f)	0.4 ± 0.6 mL/min	0.14 ± 0.16 mL/min	Significantly lower stimulated parotid salivary flow rates in HD patients (*p* = 0.01).
Kho et al. (1999) [26]	Republic of Korea	HD (22), 34.7 ± 10.8 y (64% m, 36% f) C (22), 30.5 ± 7.9 y (sex not reported)	0.67 ± 0.34 mL/min	1.11 ± 0.43 mL/min	Significantly lower stimulated parotid salivary flow rates in HD patients (*p* < 0.001).

SFR = salivary flow rate HD = patients with kidney failure on hemodialysis C = healthy controls.

## Data Availability

The scientific articles included in this review are available on request from the corresponding author.

## Appendix A

### Appendix A.1. Search Strategy Details

#### Appendix A.1.1. Pubmed Search

(“xerostomia” [mesh] OR “xerostomia” [tiab] OR “salivary” [tiab] OR “saliva” [tiab] OR “salivation” [tiab] OR “hyposaliv*” [tiab] OR “dry mouth” [tiab] OR “mouth dryness” [tiab] OR “asialia” [tiab] OR “oral dryness” [tiab]) AND (“dialysis” [tiab] OR “hemodialysis” [tiab] OR “haemodialysis” [tiab] OR “dialysing” [tiab] OR “hemodialysing” [tiab] OR “haemodialysing” [tiab] OR “hemodialyzed” [tiab] OR “haemodialyzed” [tiab] OR “dialyzed” [tiab] OR “kidney replacement therapy” [tiab] OR “renal replacement therapy” [tiab] OR “renal dialysis” [mesh]).

#### Appendix A.1.2. Embase Search

(‘xerostomia’/exp OR ‘xerostomia*’: ti, ab, kw OR ‘salivary’: ti, ab, kw OR ‘saliva’: ti, ab, kw OR ‘salivation’: ti, ab, kw OR ‘hyposaliv*’: ti, ab, kw OR ‘dry mouth’: ti, ab, kw OR ‘mouth dry*’: ti, ab, kw OR ‘dryness of the mouth’: ti, ab, kw OR ‘asialia’: ti, ab, kw OR ‘oral dryness’: ti, ab, kw) AND (‘dialysis’/exp OR ‘hemodialysis’/exp OR ‘dialysis’: ti, ab, kw OR ‘hemodialysis’: ti, ab, kw OR ‘haemodialysis’: ti, ab, kw OR ‘dialysing’: ti, ab, kw OR ‘hemodialysing’: ti, ab, kw OR ‘haemodialysing’: ti, ab, kw OR ‘dialyzed’: ti, ab, kw OR ‘hemodialyzed’: ti, ab, kw OR ‘haemodialyzed’: ti, ab, kw OR ‘renal replacement therapy’/exp OR ‘renal replacement therapy’: ti, ab, kw OR ‘kidney replacement therapy’: ti, ab, kw).

#### Appendix A.1.3. Web of Science Search

(TS = (Xerostomia* OR salivary OR saliva OR salivation OR hyposaliv* OR dry-mouth OR mouth-dry* OR dryness-of-the-mouth OR asialia OR oral-dryness)) AND (TS = (dialysis* OR hemodialysis OR heamodialysis OR dialysing OR hemodialysing OR haemodialsying OR hemodialyzed OR haemodialyzed OR dialyzed OR kidney-replacement-therapy OR renal-replacement-therapy)).
